# Physics of band-filling correction in defect calculations of solid-state materials†

**DOI:** 10.1039/d4ra01528b

**Published:** 2024-06-04

**Authors:** Harshan Reddy Gopidi, Lovelesh Vashist, Oleksandr I. Malyi

**Affiliations:** a Centre of Excellence ENSEMBLE^3^ Sp. z o. o. Wolczynska Str. 133 01-919 Warsaw Poland; b Qingyuan Innovation Laboratory Quanzhou 362801 P. R. China oleksandrmalyi@gmail.com

## Abstract

In solid-state physics/chemistry, a precise understanding of defect formation and its impact on the electronic properties of wide-bandgap insulators is a cornerstone of modern semiconductor technology. However, complexities arise in the electronic structure theory of defect formation when the latter triggers partial occupation of the conduction/valence band, necessitating accurate post-process correction to the energy calculations. Herein, we dissect these complexities, focusing specifically on the post-process band-filling corrections, a crucial element that often demands thorough treatment in defect formation studies. We recognize the importance of these corrections in maintaining the accuracy of electronic properties predictions in wide-bandgap insulators and their role in reinforcing the importance of a reliable common reference state for defect formation energy calculations. We explored solutions such as aligning deep states and electrostatic potentials, both of which have been used in previous works, showing the effect of band alignment on defect formation energy. Our findings demonstrate that the impact of defect formation on electronic structure (even deep states) can be significantly dependent on the supercell size. We also show that within band-filling calculations, one needs to account for the possible change of electronic structure induced by defect formation, which requires sufficient convergence of electronic structure with supercell size. Thus, this work emphasizes the critical steps to accurately predict defect formation energy and paves the way for future research to overcome these challenges and advance the field with more efficient and reliable predictive models.

## Abstract 2

Defect formation in insulators can result in supercell size dependence of band filling. This paper discusses an in-depth understanding and practical guidance of how such band-filling corrections should be accounted for in the defect calculations.

## Graphical abstract

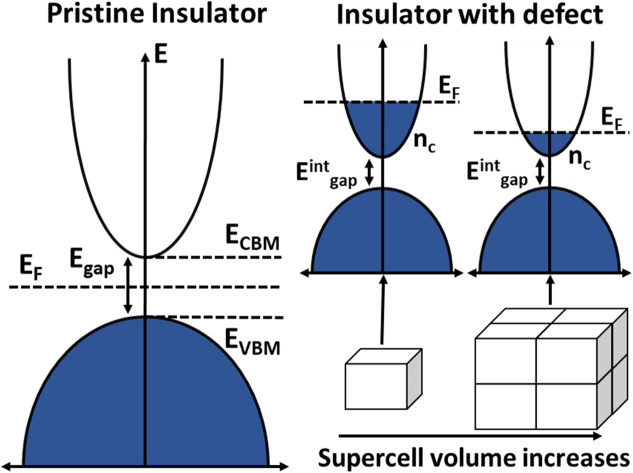

## Introduction

Point defects are present in all materials as their formation arises from a balance between the energy cost needed for their formation and the configuration entropy gain due to increased defect concentration.^1–3^ Despite being present only in low concentrations under normal conditions in most materials, defects often significantly influence the properties of materials, such as color, equilibrium Fermi level, and doping response.^4–8^ With recent advancements in electronic structure theory, defect physics and hence materials properties can be accurately predicted using first-principles calculations. However, it is important to note that density functional theory (DFT) is still usually limited to supercell calculations of only a few hundred atoms. Hence, when computing defect properties for typical supercells, the first-principles results should be extrapolated to the dilute limit. This can be achieved by either (i) scaling corresponding properties (*e.g.*, vacancy formation energy) for different defects as a function of supercell size^9–12^ or (ii) applying post-process corrections.^13–17^ While the former approach is theoretically exact and only requires sufficient supercomputer resources, it can be challenging to apply for many defects due to the need to scale each defect individually. In contrast, the latter approach typically involves applying post-process corrections to extrapolate the limit of defect formation energy and is becoming increasingly popular in modern research.

Previous works^13–19^ have shed some light on post-process corrections, and several post-process correction codes have been developed to automate defect calculations. Thus, it becomes clear that the formation energy of a point defect in an insulator is directly affected by the formation energies of other charged defects, which are a function of the parametric Fermi level. Hence, the calculation of equilibrium defect concentration or equilibrium Fermi level corresponding to given environmental conditions requires a self-consistent solution that follows the charge neutrality^4,5,20,21^ rule and accounts for the effect of the defect on the average electrostatic potential as well as the accounting for energy correction due to periodic interaction between point defects. Moreover, dielectric screening, which can vary anisotropically along different directions, must be considered when dealing with charged point defects.

**Fig. 1:**
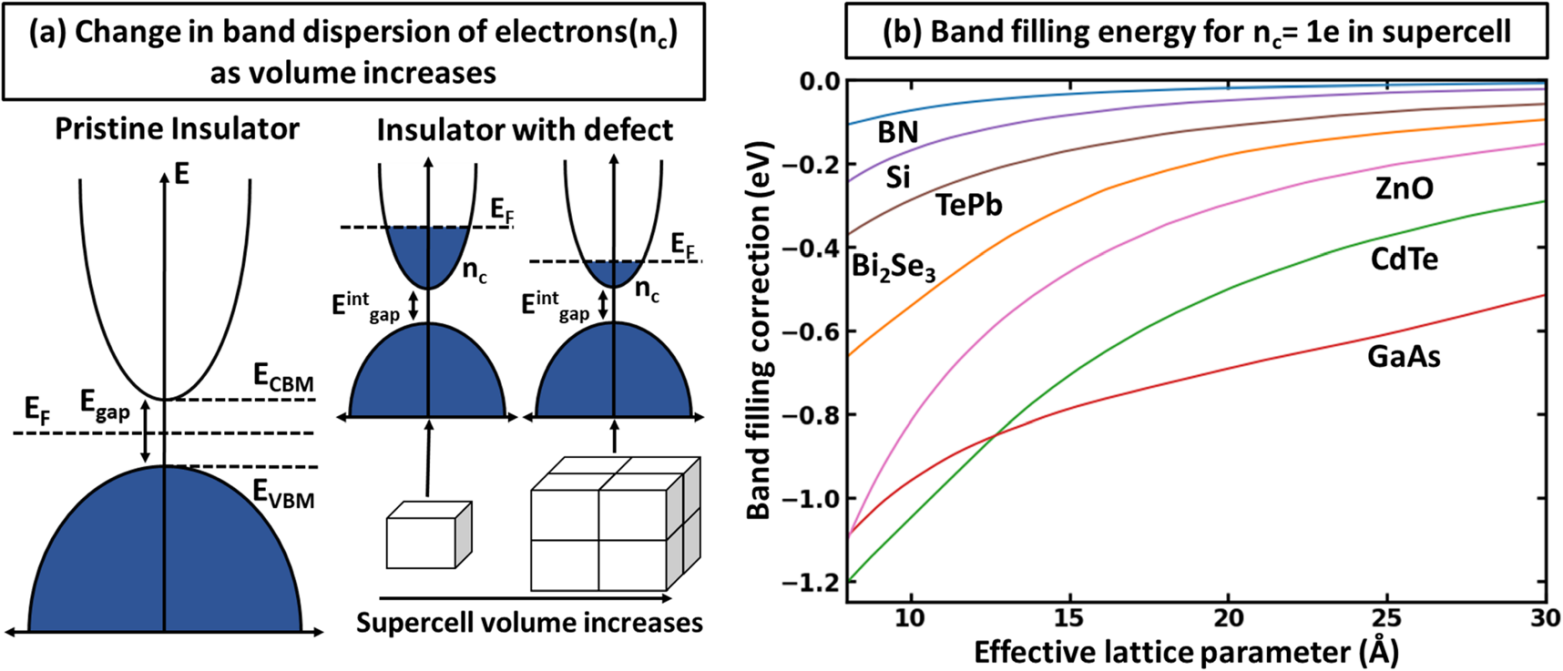
Defect formation resulting in the supercell size dependence of band filling correction. (a) Origin of band-filling correction (as a consequence of the finite size of the system). (b) Band filling correction to supercells for different materials as the function effective lattice parameter (*i.e.*, defined as the cubic root of the system volume), calculated with the rigid shift of the Fermi level in the conduction band for some well-known insulators.

There is a common misconception that post-process corrections are only necessary when dealing with charged defects and that uncharged defects do not require such corrections. This is only partially accurate. For instance, both charged and uncharged point defects can cause partial occupation of the conduction or valence band. Within electronic structure theory for a finite supercell size, such electrons or holes are spread in energy range. In contrast, in the dilute limit (one which we often aim to represent within electronic structure theory), they are expected to only contribute to the tip of band edges (see Fig. 1a). This thus implies that formation energy of point defect adding free carriers to the system requires post-process energy corrections that accounts for additional energy cost corresponding to band-filling. This additional correction for the doped insulator can be calculated by summing over the eigenvalues^14,16,18^ as:

For n-type dopants:1
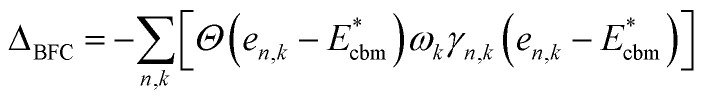


For p-type dopants:2
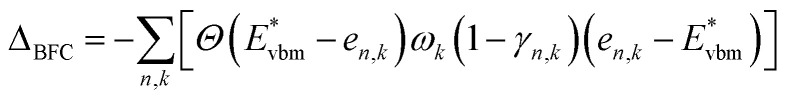
where *Θ*(*x*) is the Heaviside step function, *ω*_*k*_ are the weights of the *k*-points, *e*_*n*,*k*_ are the energy eigenvalues of state (*n*, *k*), *γ*_*n*,*k*_ are the occupations of the eigenstate (*n*, *k*), 
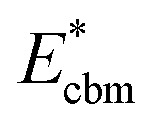
 is the aligned conduction band minimum (CBM) of supercell without defects, 
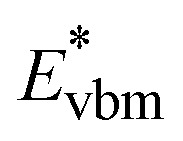
 is the aligned valence band maximum (VBM) of the supercell without defects. While some papers in the literature touch upon band-filling correction, most are anchored in exploring defect physics specific to particular compounds or simply providing eqn (1) and (2). Moreover, while thousands of papers reported defect calculations for different systems, only very small fractions of those works accounted for band-filling correction correctly. Because of this, our objective is to bridge this knowledge gap by presenting a thorough discussion and analysis of the topic. In this way, we revisit the fundamentals of band-filling correction and establish a foundational understanding of it. We elucidate the alignment of band edges relevant to band-filling correction, emphasize situations where it is crucial, offer extended methodological advice, and outline its principal limitations. This endeavor is accomplished through detailed first-principles calculations across a spectrum of different insulators, unveiling the intricacy of band-filling calculations, elucidating why eqn (1) and (2) cannot be directly employed in defect calculations, and steering how the band-filling correction ought to be accurately executed within the DFT calculations. Through this approach, we furnish not only methodological guidance but also delineate factors that may influence outcomes, thereby elucidating the physics underlying band-filling correction.

## Results and discussion

### Band-filling correction within the rigid shift model

To illustrate the need for accurate accounting of band-filling correction, in Fig. 1b, we show the expected band-filling correction due to the rigid shift of the Fermi level in the conduction band for some well-known insulators. In such an approximation, we directly use eqn (1) and calculate the band-filling correction using dense *k*-point sampling and estimating the carrier concentration corresponding to the addition of a single electron to the supercell of a given size. Here, the number of electrons (*n*_e_) at a given parametric *E̲*_F_ is calculated as 
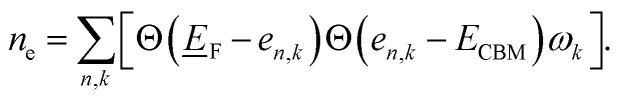
 The results demonstrate that band-filling correction is highly sensitive to the free carrier concentration and differs for different materials. One of the key points here is that an increase in band dispersion (often simply characterized by low effective mass) increases the expected band-filling correction. As an illustration, for an effective lattice parameter (defined as the cubic root of the system volume) of around 10 Å, the magnitude of band-filling correction for the system containing 1e in the conduction band is around 1 eV for CdTe, ZnO, and GaAs. At the same time, the corresponding value for BN is less than 0.1 eV. Another important conclusion is that even for very large supercells (over 20 Å), the band-filling correction is larger than 0.3 eV for a range of systems. For instance, for GaAs, the expected band-filling correction is about 0.5 eV for an effective (lattice) parameter of 30 Å. These results demonstrate that ignoring band-filling correction can completely change the defect picture of the entire compound, resulting in unphysical predictions, especially taking into account that many defects can add more than a single electron/hole to the compound. Unfortunately, since the electronic structure of real material is usually not characterized by a simplified parabolic form in the wide energy range, it is not possible to define a simplified expression correlating band-filling correction directly to the effective mass. It should also be noted that results herein are presented for Perdew–Burke–Ernzerhof (PBE)^22^ exchange-correlation (XC) functional, which tends to underestimate the band gap energy compared to experimental data.^23,24^ This underestimation can directly affect defect properties.^25,26^ However, the focus of this work is not to attain precise calculations of defect formation energies but to develop a deeper understanding of the band-filling correction and practical insight on accounting for such correction in defect calculations. Even with the band gap underestimation issue inherent to the PBE functional, it provides a reasonable qualitative picture of the electronic structure sufficient for studying band-filling corrections. In this context, the absolute value of the band gap is less important than the relative positions of the energy levels and the behavior of the electrons within them. However, in general, for a specific compound, the effective mass is sensitive to the choice of XC functional.^27–30^ Therefore, the absolute value of the band-filling correction (but not the main physics presented in this work) may also be affected as a result.

### Band-filling correction beyond the rigid shift model

**Fig. 2:**
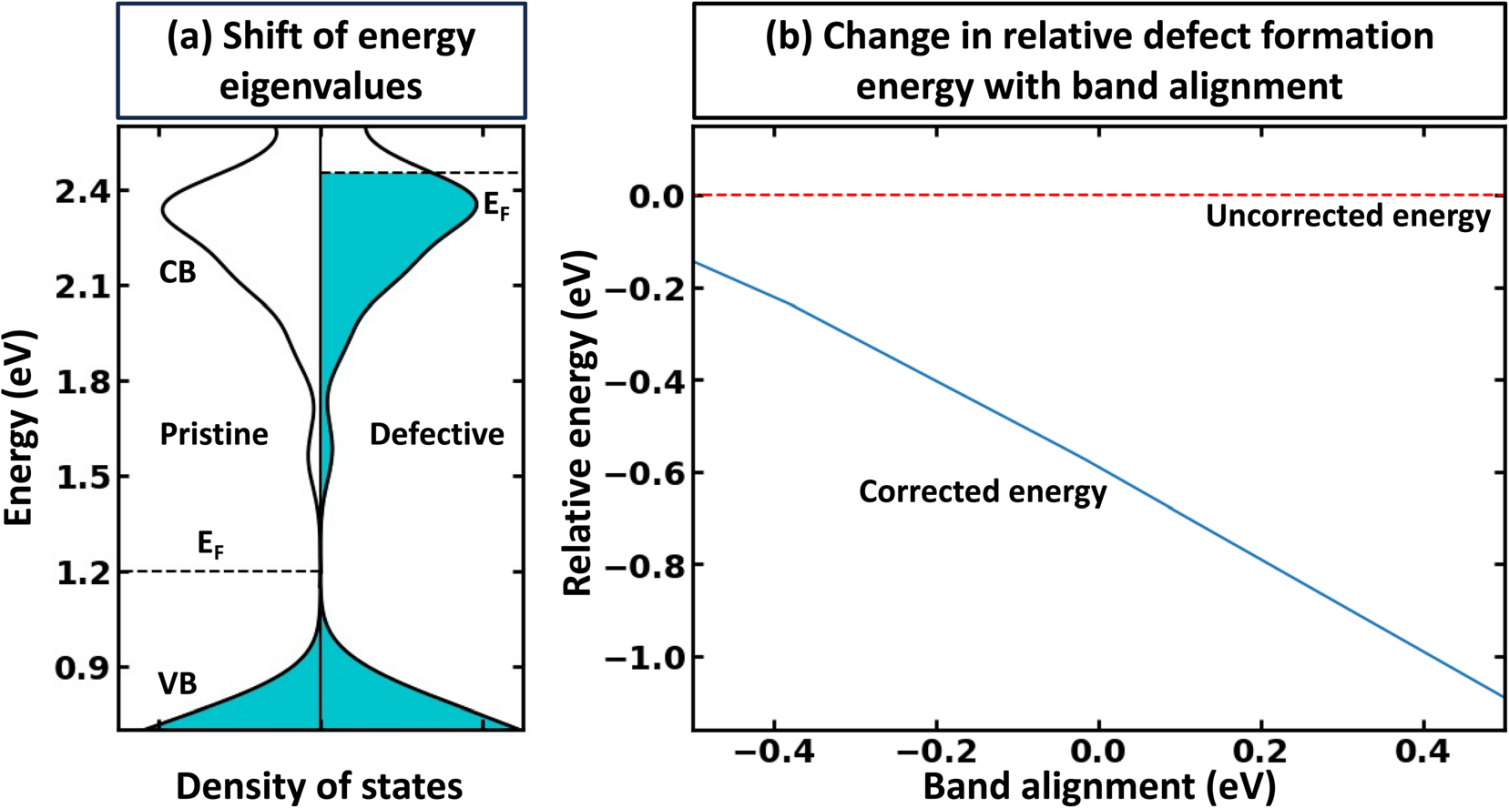
Effect of defect formation on the electronic structure of ZnO. (a) Electronic structure of pristine ZnO and defective ZnO:Al_Zn_. The defect formation results in a slight upward shift of energy eigenvalues. Occupied states are represented with shading, while unoccupied states are indicated with white. The Fermi level for the pristine system is shown in the middle of the band gap. For visualization purposes, the density of states for the principal conduction band is multiplied by 10. (b) Relative defect formation energy for Al substitutional defect (ZnO:Al_Zn_) as a function of band alignment used for calculations of band filling correction. The results are shown for PBE calculations, including band-filling correction for a 192-atom supercell.

The rigid band approximation represents an ideal scenario, typically unattainable in common materials. Indeed, in many cases, doping can result in a reactive response or a noticeable change in electronic structure.^31,32^ Hence, in practice, one must deal with materials response to doping in the self-consistent energy minimization picture. Unfortunately, eqn (1) and (2) cannot be directly used because modern first principles codes (*e.g.*, VASP^33–36^ and Quantum Espresso^37–39^) do not use common reference energy but provide direct Kohn–Sham-eigenvalues for a set of *k*-points. To illustrate this point, we examine the case of defect formation in ZnO – a conventional example of a wide band gap insulator (Fig. 2). Our findings suggest that defect formation results in the shift of the absolute energies of eigenvalues. Consequently, comparing eigenvalues for pristine and defective systems becomes meaningless. Moreover, the shift of absolute eigenvalues differs for different compounds, defects, and supercells, suggesting that eqn (1) and (2) mentioned above necessitates the use of a common reference state. Such a common reference state, however, should distinguish, for instance, the shift of Kohn–Sham-eigenvalues due to a non-common reference state as well as change in electronic properties caused by the formation of the defect (*e.g.*, due to localized structural displacements or change local bonding environments). Hence, one cannot directly align the valence/conduction bands for defective and pristine systems. For example, Fig. 2a demonstrates the electronic density of states for pristine and defective ZnO systems, revealing a clear shift in eigenvalue energies between the two systems. Thus, it can be inferred that comparing the electronic structures of different systems is only meaningful when a common reference state is defined, thus making band alignment very challenging and an essential step of calculations for band-filling correction. The sensitivity of defect formation energy to band alignment (where it is treated as a parametric value) is shown in Fig. 2b. The question then arises as to how we define the common reference state. The answer is not straightforward and is contingent upon the specifics of the system under examination, which we will discuss further.

### Alignment of deep states (average) as the reference state for band-filling correction

**Fig. 3:**
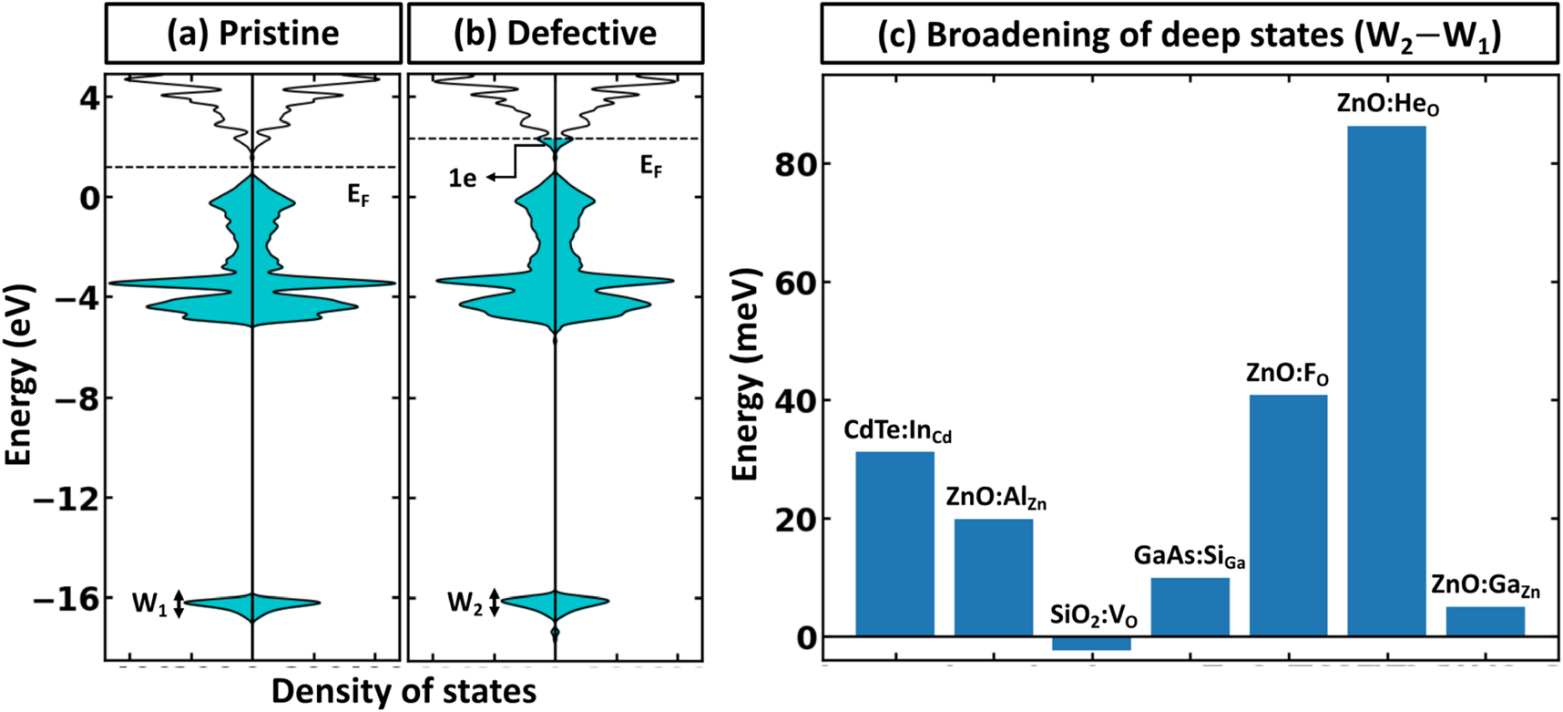
Effect of defect formation on the deep states. Density of states of (a) pristine and (b) defective ZnO:Al_Zn_ systems. The results are shown for a 192-atom supercell. For visualization purposes, the density of states for the principal conduction band is multiplied by 30. The Fermi level for the pristine system is shown in the middle of the band gap. (c) Histogram for broadening of deep states (defined here as deeper than 10 eV from the principal valence band in the pristine system) upon defect formation. The results are presented using PBE for 54, 192, 162, 54, 96, 96, and 96-atom supercells for CdTe:In_Cd_, ZnO:Al_Zn_, SiO_2_:V_O_, GaAs:Si_Ga_, ZnO:F_O_, ZnO:He_O_, and ZnO:Ga_Zn_, respectively.

Within the global scientific community, there is recognition of the significant role that the reorganization of valence states plays in the formation and nature of chemical bonds. The foundation of this principle is based on the idea that transitions in the electron states of an atom's outermost shell do not simply dictate the variety of bonds that the atom is capable of forming. In addition, these shifts also wield a considerable influence on key factors such as the strength, stability, and overall reactivity of the bonds themselves. Because of this, core electrons or, more generally, deep states, which occupy lower energy levels and are somewhat insulated by the outer electron shell from direct influences of other atoms (they still indirectly influence bonding) can be used as potential consistent reference points across various systems to compare the electronic structure of different systems.^40^ In the case of full electron codes (*e.g.*, Wien2k^41^), one can explicitly calculate the position of the deepest orbitals and align them to define the common reference state. We note, however, that the most widely used first-principles codes use the pseudopotentials^42–44^ to simplify the computational process and reduce the computational cost associated with solving the Kohn–Sham equation.^45^ As a result, the position of the deep state is only 10–25 eV below the Fermi level, depending on the considered valence configuration. Because of this, such states can often be affected by local structural/bonding changes. In Fig. 3a and b, we summarize the effect of defect formation on the deep states for a few representative compounds and supercells without any relaxation for the defective systems. These results show that even deep states can have substantial broadening. For instance, for CdTe:In_Cd_ described within a 54-atom supercell, the broadening is 31 meV (Fig. 3c). It should also be noted that defect formation can, in principle, cause interference of defect-deep states with the states of the host atoms as seen in Fig. 3b. One might initially think that a small change or broadening of the deep states would not significantly affect the defect formation energy. However, as shown in Fig. 2b, the band alignment does directly affect the results, and the effect is expected to increase with higher free carrier concentration introduced by the defects. This means that accurate defect calculations will require not only accounting of band-filling correction but also an accurate alignment of energy states of pristine and defective systems. It is also probable that for many such systems, one cannot solely rely on utilizing modern pseudopotential-based DFT codes to align deep states (at least without including spatial dependence of energy for such states).

### Alignment of electrostatic potentials as reference state for band-filling correction

**Fig. 4:**
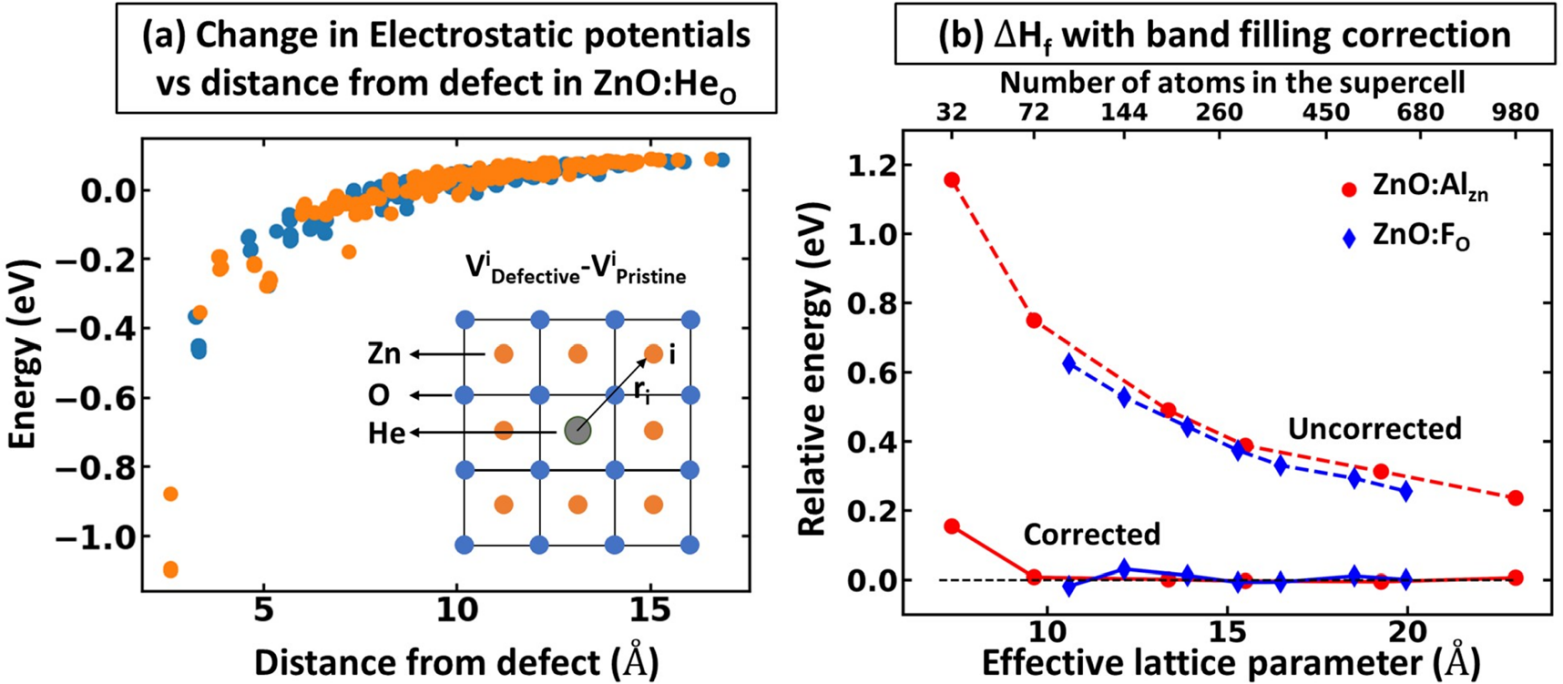
Electrostatic potential as reference state for band alignment. (a) Change in electrostatic potentials (*V*_defective_*i* − *V*_pristine_*i*, where *V*_defective_*i* and *V*_pristine_*i* are the electrostatic potentials in defective and pristine systems, respectively) as a function of the distance (in defective supercell) from the defect shown in the example of ZnO:He_O_ using a 640-atom supercell. (b) Supercell size dependence of relative defect formation energy for ZnO:Al_Zn_ and ZnO:F_O_. For ZnO:F_O_ and ZnO:Ga_Zn_ (not shown), the results are visually not distinguishable. The results are shown for PBE calculations, including band-filling correction using energy alignment from most remote Zn/O atoms in the system (the calculation is based on the average fluctuation in electrostatic potentials for atoms positioned within spherical shells defined by two radii, *r*_max_ and *r*_max_ − *r*_tol_, here, *r*_max_ represents the maximum radial distance of any atom within the supercell from the defect, while *r*_tol_ is a tolerance radius set to be 2 Å).

While defects can significantly influence the electronic properties of materials – given that these properties are highly sensitive to specific atomic arrangements, bonding situations, and shifts in the local environment^28,46–48^ – the average electrostatic potentials at cores, as calculated by VASP, within the system, remain comparatively undisturbed. This stability stems from the fact that electrostatic potential is a long-range attribute derived from charge interactions spanning vast distances. Consequently, the effects of large-scale environmental shifts are averaged out, while the impact of the local environment remains crucial. As one moves sufficiently far from the defect, the local environments mirror that of the pristine system. In such regions, comparing the electrostatic potentials offers insight into energy alignment. Consequently, it is viable to use the mean electrostatic potentials – particularly, potentials that are spherically averaged around atoms within a specified distance – for two systems as a common method for energy alignment. This strategy is particularly applicable when calculating defect formation energy. However, we note that introducing the defect inside the supercell results in symmetry breaking, making different atoms inequivalent to each other and resulting in the space and atomic identity-dependent electrostatic potentials. This is shown in Fig. 4a, where one can see that the change in relative electrostatic potential slowly converges with moving away from the defect, reaching the constant value (within 0.01 eV) at about 15 Å away from the defect, as shown by an example of ZnO:He_O_ system (herein, modeled as 640-atom supercell)- a type of functional defect resulting in putting Fermi level to the conduction band with 2e spread over a wide energy range in the conduction band.^49,50^ We also note that the tolerance value for band alignment needed for accurate calculation of defect formation energy differs for different systems as the band filling correction is affected by the band alignment and free carrier concentration. In other words, the large free carrier concentration (intrinsic or doping induced) requires more accurate band alignment calculations. While the results are demonstrated on example ZnO, as noted in Fig. 1, the effect is expected to be present for all compounds. To show the power of band-filling correction, we consider ZnO:Al_Zn_, a system where the formation of the point defect (Al on Zn site) puts 1e in the conduction band. Indeed, Al is one of the standard n-type dopants in ZnO to realize n-type transparent conductive oxides.^51–53^ The uncorrected PBE defect formation energy has a strong supercell size dependence of defect formation energy (Fig. 4b), with relative defect formation energy changing by 0.5 eV as supercell size increases from 72 to 980 atoms. Importantly, when band-filling correction is accounted for (accounting for energy alignment using an electrostatic potential for atoms most remote from the defect), the defect formation energy becomes almost supercell independent. These results thus demonstrate the power of band-filling correction to eliminate a significant part of supercell size dependence of defect formation energy. Moreover, similar to the rigid shift model, we show that even for 980-atom supercell, there is sufficient large band filling correction (over 0.2 eV).

### Other factors that should be accounted for in the post-process correction

**Fig. 5:**
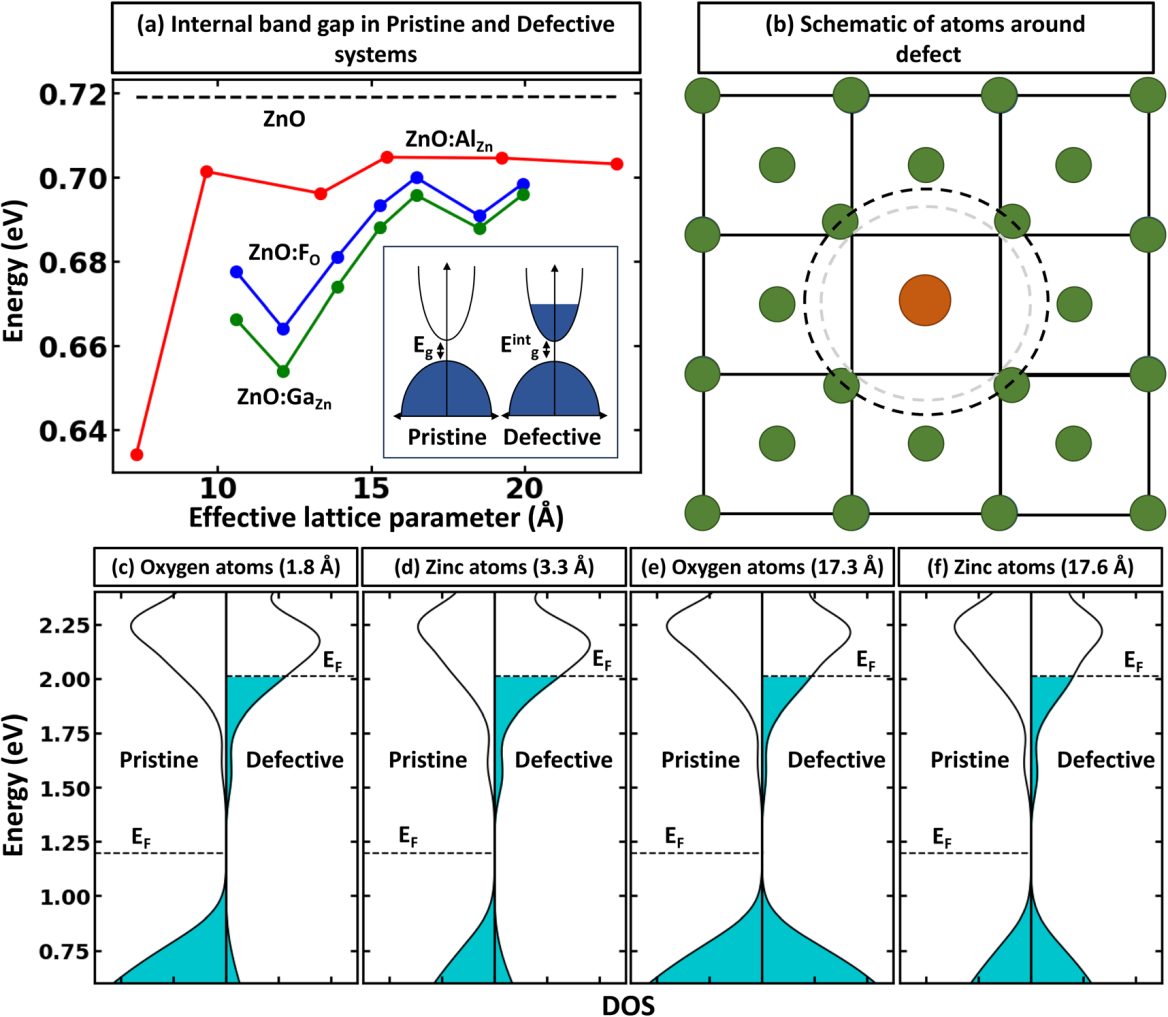
Other factors that should be accounted for the post-process correction. (a) Illustrates the variation in the band gap as a function of supercell size, demonstrating the material response to supercell size. The results are shown for ZnO:Al_Zn_, ZnO:F_O_, ZnO:Ga_Zn_ and ZnO supercells. (b) Shows schematically the local perturbations of atoms near the defect. Panels (c) to (f) represent the projected density of states, after band alignment, at different distances from the defect position in ZnO (on the left) and ZnO:Al_Zn_ (on the right) supercells comprising 980 atoms. These panels provide insights into the electronic state distribution in various atomic spheres of the system. The Fermi level for the pristine system is shown in the middle of the band gap. For visualization purposes, the projected density of states for the principal conduction band are multiplied by 40.

We must recognize that the lack of dependence of defect formation energy on the supercell size, even after considering the post-process band-filling correction, does not necessarily stand as a complete representation of accurately depicted primary physics. Eqn (1) and (2) take into account the effect of band-filling on the kinetic energy of a system composed of non-interacting particles within the context of Kohn–Sham DFT. However, this method does not account for the relaxation or displacement convergence relative to the supercell size. For certain systems, this particular aspect may be profoundly significant. This is well illustrated in the scenario of noble gas functional defects that exhibit atypical relaxation patterns in solids. For instance, achieving convergence of atomic displacements in noble gas-doped ZnO often necessitates the use of a supercell comprised of more than 500 atoms.^50^ Moreover, it is crucial to acknowledge that the correction detailed in Eqn (1) and (2) only extends to the band edge of the pristine system. Therefore, if the defect formation results in alterations of the internal band gap, the band-filling correction will not account for these changes. Let us understand the latter case in detail. When a defect is introduced, it breaks the internal symmetry. This symmetry breaking may modify the local density of states for the nearest atoms significantly, resulting in a change in the overall internal band gap energy. For instance, Fig. 5a demonstrates the convergence of the PBE internal band gap between principal band edges of ZnO:Al_Zn_ as a function of supercell size with atom projected density of states. Here, one can see two main points: (i) there is supercell size dependence of internal band gap; (ii) the defected system has an internal gap between principal band edges smaller than the undoped system in the dilute limit (infinite supercell). It is critical to note that the reduction of band gap energy in the dilute limit can be a fully physical phenomenon originating from the formation of different motifs contributing differently to electronic properties. This, indeed, is a common phenomenon in the case of symmetry breaking in quantum materials.^28,46^ From Fig. 5b–f, it is evident that the principal valence band sensitivity is significantly influenced by the proximity to the defect. As one moves further from the defect, the local environment increasingly resembles the electronic structure of pristine ZnO. Such behavior underscores the profound impact of the Al defect on the system and the origin for the change of internal band gap energy. However, the change in electronic properties can also be an example of artificial interaction between the defect-induced local environment and its periodic images. Thus, as shown in Fig. 5a, at the small size of supercells, the internal band gap energy can be substantially smaller than the asymptotic band gap energy. These results thus demonstrate that while band-filling correction can account for artificial filling of the conduction band, additional correction may need to be used when the electronic structure or atomic displacements are not converged with supercell size.

## Conclusions

This study highlights the necessity of applying post-process band-filling corrections in defect energy calculation when defects trigger partial occupation of the conduction or valence band. We underline the need for a robust, common reference state (energy alignment) in defect formation energy calculations, which becomes challenging given the local perturbation of structure and potential changes to the internal band gap upon defect formation. We have explored possible solutions, including aligning deep states and electrostatic potentials (which are used in some of the previous works), but have also highlighted the inherent difficulties in each approach. We have also pointed out that the impact of defect formation on electronic properties can be notably supercell size-dependent in some cases. This study thus serves as an important stepping stone towards a more comprehensive understanding of defect formation and electronic structure theory. It underscores the urgency of developing more accurate, encompassing methodologies to better predict and account for these dynamics in the context of varying supercell sizes and defect-induced alterations. Future research should tackle these challenges to advance the field and realize more efficient and reliable predictive models.

## Methods

### First-principles calculations

All calculations at the first-principle level were performed using the Vienna *Ab initio* Simulation Package (VASP),^33–36^ employing the PBE^22^ XC functional. For plane wave basis, the cutoff energy levels were set to 520 eV and 500 eV for volume relaxation (carried out only for primitive undoped cells) and structural relaxation, respectively (except for ZnO:F_O_, ZnO:He_O_ and, ZnO:Ga_Zn_ calculations of volume and structural relaxations, if performed, are done with 550 and 450 eV respectively). The atomic relaxations were carried out (unless otherwise specified) until the intrinsic forces were below 0.01 eV Å^−1^. The Γ-centered Monkhorst–Pack k-grid^54^ with the density of 10 000 per reciprocal atom was used for all main calculations (which is sufficient to capture the main physics of the band-filling correction). The results were analyzed using Pymatgen^55^ and Vesta.^56^

### Defect calculations

To study the defect formation energy in the dilute limit, only one defect is created per supercell and the lattice parameters of supercells were fixed for defect relaxations. The defect formation energy was calculated as
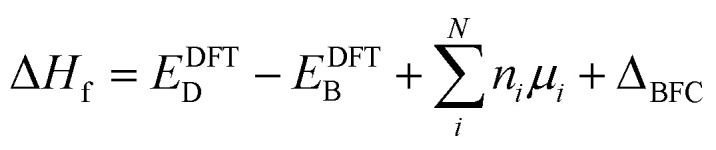
where *E*^DFT^_D_ and *E*^DFT^_B_ are the energy of defective and pristine systems, respectively; *n*_*i*_ is the number of atoms of type *i* added/removed (if removed, *n*_*i*_ > 0; if added, *n*_*i*_ < 0) to the system. *μ*_*i*_ is the chemical potential of atom type *i*. *Δ*_BFC_ is the band filling correction (this term is excluded when we present uncorrected defect formation energy). The primary focus of our study is to elucidate the impact of band-filling correction rather than to determine the exact value of defect formation energy. Because of this and since for a given type of defect 
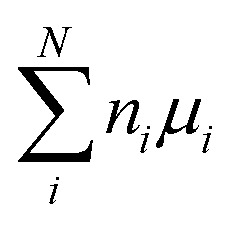
 is a constant, we present results for relative defect formation energy, which is independent on the chemical potential term.

Alignment using the electrostatic potentials (ESP) is performed by aligning the averages of electrostatic potentials of most remote atoms in the system. The calculation is based on the average fluctuation in electrostatic potentials for atoms positioned within spherical shells defined by two radii, *r*_max_ and *r*_max_ − *r*_tol_, here, *r*_max_ represents the maximum radial distance of any atom within the supercell from the defect, while *r*_tol_ is a tolerance radius set to be 2 Å. The formula for the potential alignment using ESP (*V*^ESP^_pa_) is be given by:
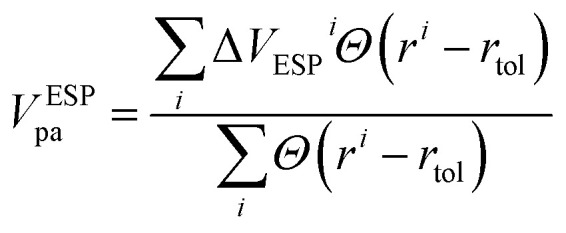
where *Θ* is the Heaviside step function, Δ*V*_ESP_^*i*^ is the change in electrostatic potential of atom with index *i*, after the introduction of the defect. *r*^*i*^ is the distance of atom with index *i* from the defect.

## Data availability

The data that support the findings of this study are available from the corresponding author upon reasonable request.

## Conflicts of interest

The authors have no conflicts to disclose.

## Supplementary Material

RA-014-D4RA01528B-s001
