# Supplementary material for: Physics of band-filling correction in defect calculations of solid-state materials
Source: RSC Adv. 2024 Jun 4;14(25):17675–83. doi: 10.1039/d4ra01528b (PMC11148636; doi:10.1039/d4ra01528b)
Supplement: RA-014-D4RA01528B-s001 [file RA-014-D4RA01528B-s001.pdf]

Electronic Supplementary Material (ESI) for RSC Advances.
This journal is © The Royal Society of Chemistry 2024

Supporting Information
for
Physics of band-filling correction in defect calculations of solid-state materials

Harshan Reddy Gopidi[1], Lovelesh Vashist[1], and Oleksandr I. Malyi[,2,1#]

[1]Centre of Excellence ENSEMBLE[3] Sp. z o. o., Wolczynska Str. 133, 01-919, Warsaw, Poland

[2]Qingyuan Innovation Laboratory, Quanzhou, 362801 P. R. China

[#]**Email:** oleksandrmalyi@gmail.com

Supercell sizes used in the calculations: For results shown in Fig. 3, for CdTe:In_{Cd}, ZnO:Al_{Zn}, SiO_2:V_O, GaAs:Si_{Ga}, ZnO:F_O, ZnO:He_O, and ZnO:Ga_{Zn} systems, supercells were generated by repeating the primitive unit cell (cif file provided at the end of supplementary information) by 3×3×3 (54 atoms), 4×4×3 (192 atoms), 3×3×3 (162 atoms), 3×3×3 (54 atoms) respectively. For ZnO:F_O, ZnO:He_O, and ZnO:Ga_{Zn} systems, supercells were generated by repeating $\begin{pmatrix} 1 & -1 & 0 \\ 1 & 1 & 0 \\ 0 & 0 & 1 \end{pmatrix}$ cell (the cif file provided at the end of supplementary information) by 2×3×2 (96-atoms) for all systems.

For results shown in Fig. 4b and Fig. 5a, for the ZnO:Al_{Zn} system, supercells were generated by repeating the primitive unit cell (cif file provided at the end of supplementary information) by 2×2×2 (32 atoms), 3×3×2 (72 atoms), 4×4×3 (192 atoms), 5×5×3 (300 atoms), 6×6×4 (576 atoms), 7×7×5 (980 atoms). In ZnO:Ga_{Zn} and ZnO:F_O systems, the supercells were generated by repeating $\begin{pmatrix} 1 & -1 & 0 \\ 1 & 1 & 0 \\ 0 & 0 & 1 \end{pmatrix}$ cell (the cif file provided at the end of supplementary information) by 2×3×2 (96 atoms), 3×3×2 (144 atoms), 3×3×3 (216 atoms), 3×4×3 (288 atoms), 3×5×3 (360 atoms), 4×4×4 (512 atoms), 4×5×4 (640 atoms).

Comparison of deep-state alignment vs. alignment of average electrostatic potential as reference for band-filling correction: To compare different methods for alignment of electronic structure, we calculate band-filling correction with alignment using deep states (DS) and electrostatic potentials (ESP). Here, alignment using DS is performed by aligning the k-point weighted average of deep-state eigenvalues (of the host atoms, i.e., excluding the defect) with and without the defect, while the ESP alignment is carried out by aligning the averages of most remote atoms in the system (here, the calculation is based on the average fluctuation in electrostatic potentials for atoms positioned within spherical shells defined by two radii, r_{max} and r_{max}-r_{tol}, here, r_{max} represents the maximum radial distance of any atom within the supercell from the defect, while r_{tol} is a tolerance radius set to be 2 Å). The results show that the defect formation energy after band-filling correction, i.e., band-filling corrected energy (BFCE), converges faster with alignment using electrostatic potentials (ESP) than deep states (DS), as evident in Fig. S1-S4. Moreover, in all the systems studied, the band-filling correction is overestimated when aligned with DS in comparison to alignment with ESP.

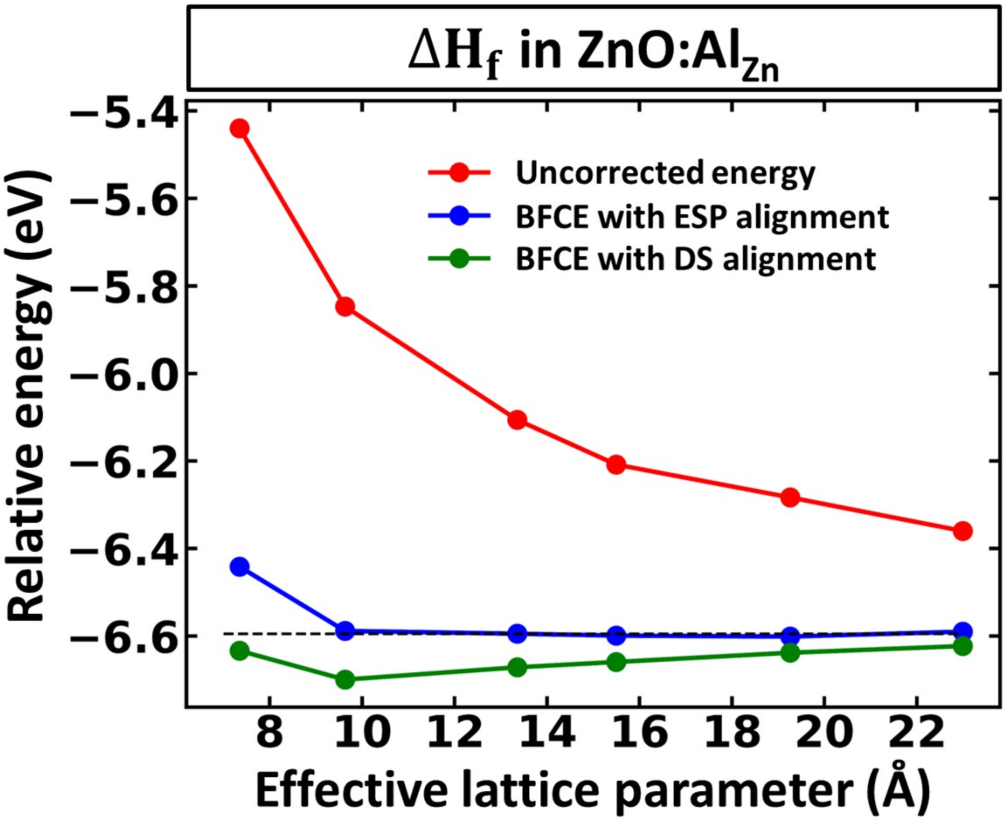

Figure S1. Relative defect formation energy (ΔH_f) vs effective lattice parameter in ZnO:Al_{Zn}. Here, the dotted line is drawn as a reference to show convergence in defect formation energy after band-filling correction with ESP alignment.

Table S1. Band filling correction energy values with supercells size (number of atoms) in ZnO:Al_{Zn}.

ZnO:Al_{Zn}							
Number of atoms		32	72	192	300	576	980
Band filling correction (eV)	ESP	-1.00	-0.74	-0.49	-0.39	-0.32	-0.23
	DS	-1.19	-0.85	-0.57	-0.45	-0.35	-0.26

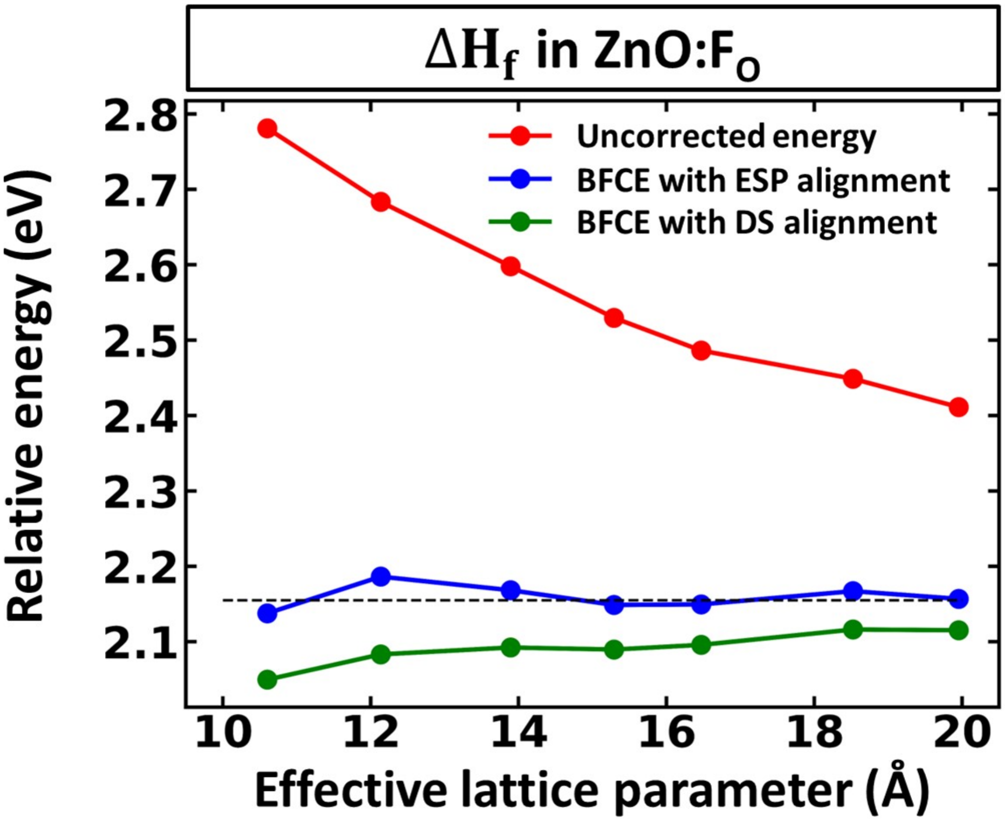

Figure S2. Relative defect formation energy (ΔH_f) vs effective lattice parameter in ZnO:F_O. Here, the dotted line is drawn as a reference to show convergence in defect formation energy after band-filling correction with ESP alignment.

Table S2. Band filling correction energy values with supercells size (number of atoms) in ZnO:F_O.

ZnO:F_O								
Number of atoms		96	144	216	288	360	512	640
Band filling correction (eV)	ESP	-0.64	-0.5	-0.43	-0.38	-0.34	-0.28	-0.25
	DS	-0.73	-0.60	-0.51	-0.44	-0.39	-0.33	-0.30

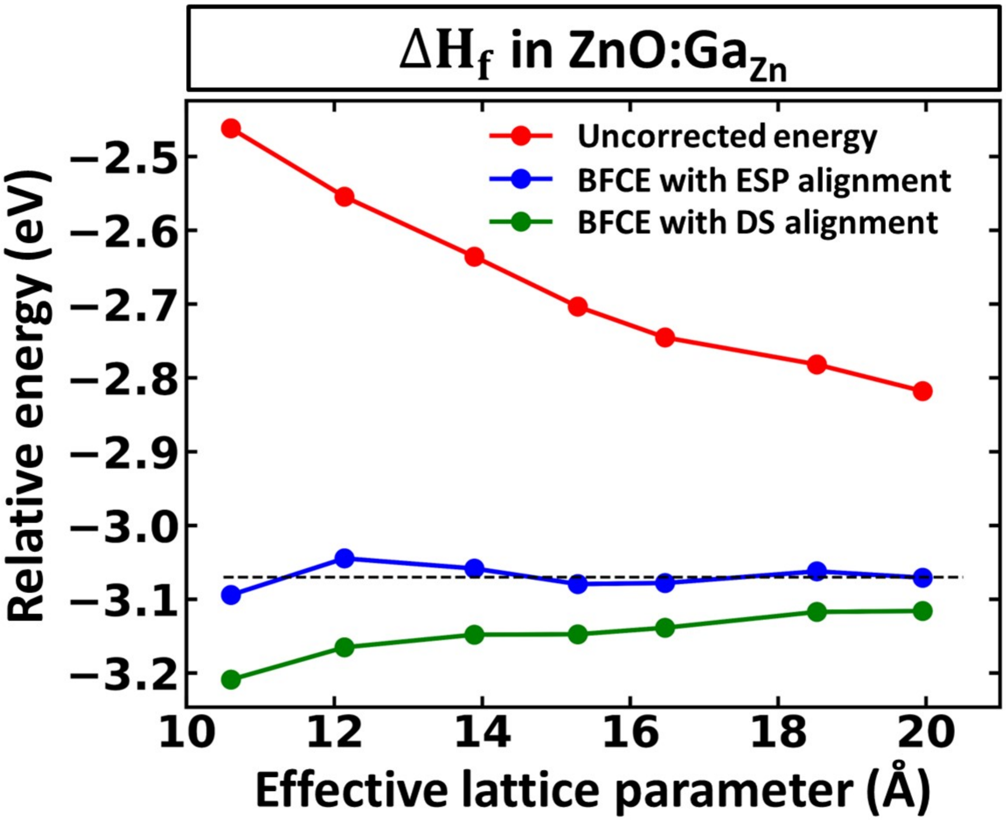

Figure S3. Relative defect formation energy (ΔH_f) with effective lattice parameter in ZnO:Ga_{Zn}. Here, the dotted line is drawn as a reference to show convergence in defect formation energy after band-filling correction with ESP alignment.

Table S3. Band filling correction energy values with supercells size (number of atoms) in ZnO:Ga_{Zn}.

ZnO:Ga_{Zn}								
Number of atoms		96	144	216	288	360	512	640
Band filling correction (eV)	ESP	-0.63	-0.49	-0.42	-0.38	-0.33	-0.28	-0.25
	DS	-0.75	-0.61	-0.51	-0.44	-0.39	-0.34	-0.30

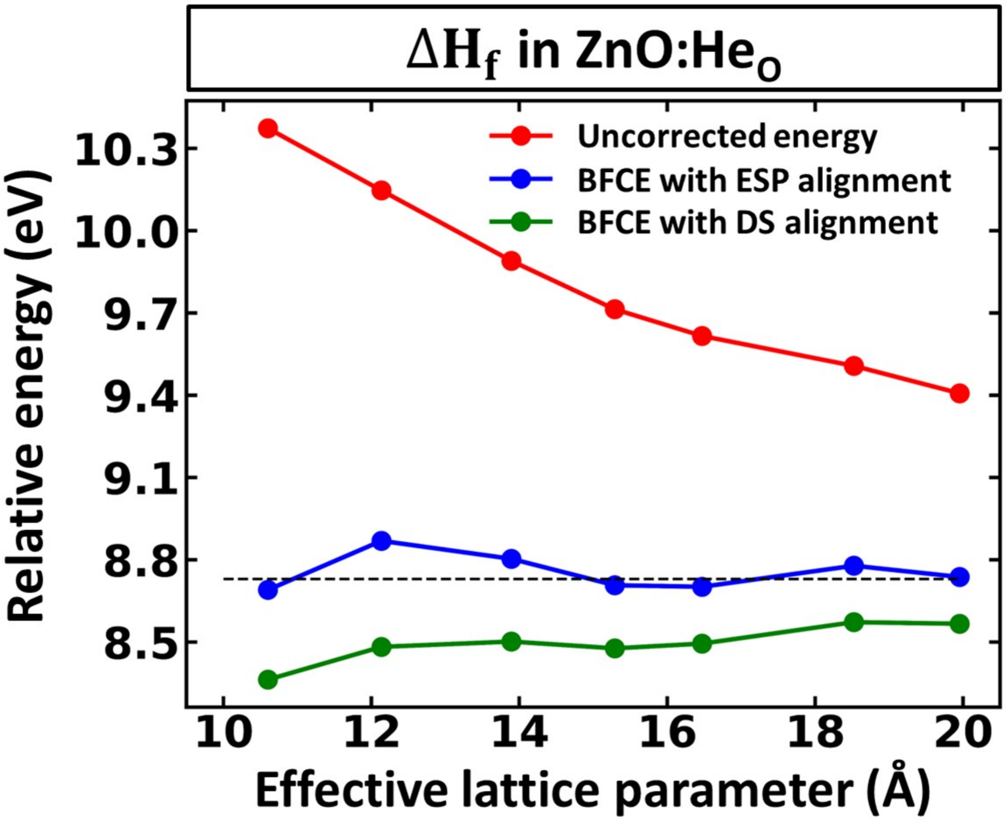

Figure S4. Relative defect formation energy (ΔH_f) with effective lattice parameter in ZnO:He$_O$. Here, the dotted line is drawn as a reference to show convergence in defect formation energy after band filling correction with ESP alignment.

Table S4. Band filling correction energy values with supercells size (number of atoms) in ZnO:He$_O$.

ZnO:He$_O$								
Number of atoms		96	144	216	288	360	512	640
Band filling correction (eV)	ESP	-1.68	-1.28	-1.09	-1.01	-0.91	-0.73	-0.67
	DS	-2.01	-1.66	-1.39	-1.24	-1.12	-0.93	-0.84

CIF file of the ZnO primitive unit cell that is used to generate the supercells of ZnO:Al_{Zn}.

```
# generated using pymatgen
data_ZnO
_symmetry_space_group_name_H-M   'P 1'
_cell_length_a   3.28725843
_cell_length_b   3.28725843
_cell_length_c   5.30305673
_cell_angle_alpha   90.00000000
_cell_angle_beta   90.00000000
_cell_angle_gamma   120.00000000
_symmetry_Int_Tables_number   1
_chemical_formula_structural   ZnO
_chemical_formula_sum   'Zn2 O2'
_cell_volume   49.62775167
_cell_formula_units_Z   2
loop_
 _symmetry_equiv_pos_site_id
 _symmetry_equiv_pos_as_xyz
   'x, y, z'
loop_
 _atom_site_type_symbol
 _atom_site_label
 _atom_site_symmetry_multiplicity
 _atom_site_fract_x
 _atom_site_fract_y
 _atom_site_fract_z
 _atom_site_occupancy
  Zn  Zn0  1  0.66666667  0.33333333  0.50057254  1
  Zn  Zn1  1  0.33333333  0.66666667  0.00057254  1
  O   O2   1  0.66666667  0.33333333  0.87973746  1
  O   O3   1  0.33333333  0.66666667  0.37973746  1
```

CIF file of the ZnO cell that is used to generate the supercells of ZnO:Ga_{Zn}, ZnO:F_O, ZnO:He_O.

```
# generated using pymatgen
data_ZnO
_symmetry_space_group_name_H-M   'P 1'
_cell_length_a   5.69627499
_cell_length_b   3.28865865
_cell_length_c   5.30069720
_cell_angle_alpha   89.99937365
_cell_angle_beta   90.00003626
_cell_angle_gamma   89.99912924
_symmetry_Int_Tables_number   1
_chemical_formula_structural   ZnO
_chemical_formula_sum   'Zn4 O4'
_cell_volume   99.29851171
_cell_formula_units_Z   4
loop_
 _symmetry_equiv_pos_site_id
 _symmetry_equiv_pos_as_xyz
  1  'x, y, z'
loop_
 _atom_site_type_symbol
 _atom_site_label
 _atom_site_symmetry_multiplicity
 _atom_site_fract_x
 _atom_site_fract_y
 _atom_site_fract_z
 _atom_site_occupancy
  O  O0  1  0.16750269  0.50346855  0.87868831  1
  O  O1  1  0.66750269  0.00346855  0.87868831  1
  O  O2  1  0.83403973  0.50353638  0.37866538  1
  O  O3  1  0.33403973  0.00353638  0.37866538  1
  Zn  Zn4  1  0.16734542  0.50355819  0.49900596  1
  Zn  Zn5  1  0.66734542  0.00355819  0.49900596  1
  Zn  Zn6  1  0.83418282  0.50345287  0.99899510  1
  Zn  Zn7  1  0.33418282  0.00345287  0.99899510  1
```
